# A comprehensive evaluation of plasma metagenomics sequencing for the diagnosis of suspected infection in pediatric patients with hematologic diseases

**DOI:** 10.3389/fcimb.2025.1584214

**Published:** 2025-05-09

**Authors:** Shihai Zhang, Qiang Guo, Wei Gai, Yuxin Guo, Yafeng Zheng

**Affiliations:** ^1^ Department of Clinical Laboratory, Anhui Provincial Children’s Hospital, Hefei, Anhui, China; ^2^ Medical Affairs Department, WillingMed Technology Beijing Co., Ltd., Beijing, China

**Keywords:** hematologic diseases, infection, cell-free DNA, metagenomic next-generation sequencing, pediatric

## Abstract

**Background:**

As a non-invasive technology, plasma cell-free DNA (cfDNA) next-generation sequencing (mNGS) has been widely used for clinical detection of a variety of infectious diseases. Infections are a major cause of poor prognosis in children with hematologic diseases. So far, there has been limited research on the use of plasma cfDNA mNGS in children with hematological disorders at high risk of infection.

**Methods:**

We retrospectively analyzed the clinical data of 73 children with hematological disorders suspected of early infection admitted to Anhui Children’s Hospital between September 2023 and February 2024. The diagnostic performance and clinical implications of mNGS versus conventional microbiological testing (CMT) were evaluated.

**Results:**

The positive rate of mNGS was significantly higher than that of CMT (69.86% vs 31.51%, *P* < 0.001). When compared with the final clinical diagnosis, the sensitivity of mNGS was significantly higher than that of CMT (71.88% vs 35.94%, *P* < 0.001). There is a high degree of agreement between the positive results of the two assays (78.95%). A total of 46 pathogens were identified in children with hematologic diseases, of which 41 pathogens were detected by mNGS and only 12 pathogens were detected by CMT. In these patients, the most common bacteria detected were *Klebsiella pneumoniae* and *Mycoplasma pneumoniae*. *Human betaherpesvirus 5* (CMV) was the most commonly detected virus. All fungi were detected only by mNGS. Overall, mNGS had a positive effect on the clinical treatment for 65.75% of patients in this study. Positive results are more likely to be obtained with mNGS when white blood cell counts, neutrophil counts, and lymphocyte counts are low.

**Conclusions:**

Early plasma cfDNA mNGS improved the performance of pathogen detection in children with hematological diseases. Rapid identification of the pathogen followed by precise targeted antimicrobial therapy improves the prognosis of patients.

## 1 Introduction

Hematologic diseases such as leukemia, lymphoma, and anemia have a high incidence in children (Grace and Ware, 2019). Deficiencies in immune function make children more susceptible to infection by a variety of pathogens. In particular, children with hematologic malignancies, especially those undergoing chemotherapy or hematopoietic stem cell transplantation (HSCT), are more likely to develop infections due to immune system damage, which is one of the leading causes of death in these children when infected (Maschmeyer et al., 2019; Zhang et al., 2023). Early intervention in children with infectious diseases can reduce the risk of death (Abe et al., 2019). However, uncertain diagnosis and treatment may lead to disease progression or death, and excessive use of inappropriate antimicrobials may lead to the emergence of multidrug-resistant bacteria, increasing the medical burden of patients (Wu et al., 2024). Therefore, it is very important to provide patients with pathogen detection and diagnosis in a timely manner so that appropriate antimicrobial agents can be used or adjusted for early targeted therapy.

Patients with blood disorders are prone to symptoms of infection such as fever after treatment (Guo et al., 2022). Early identification and exclusion of the patient’s infection is crucial. Due to the time-consuming nature of traditional testing methods and the low pathogen detection rate, children with suspected infections often receive empiric antimicrobial therapy, which can lead to antibiotic abuse (Martinez and Wolk, 2016; Martinez-Nadal et al., 2020). Although blood cultures are the gold standard for detecting blood infections in patients, blood cultures have low sensitivity and are highly influenced by antibiotic use (Wu et al., 2024). As a result, 15% to 60% of cases remain undiagnosed despite routine laboratory testing (Schlaberg et al., 2017; Xu et al., 2024).

Unbiased metagenomic next-generation sequencing (mNGS) has been used in the diagnosis of many types of infectious diseases (Blauwkamp et al., 2019; Kalbitz et al., 2024). As an auxiliary tool for routine testing, it addresses the shortcomings of conventional microbial testing (CMT), including the low positive detection rate, the time-consuming nature, and the detection bias greatly influenced by antibiotic use (Miao et al., 2018). Previous studies have focused on the performance of mNGS in adults and more in pediatric patients, with more studies of the performance of patients with hematologic malignancies (Wang et al., 2022; Feng et al., 2024; Wang et al., 2024). However, the early diagnosis and real clinical impact of mNGS on children with hematologic disorders is limited. Therefore, we evaluated the diagnostic performance and clinical impact of plasma mNGS in children with hematologic diseases in a tertiary children’s hospital in southern China to better understand the clinical application of plasma mNGS in children with hematologic diseases.

## 2 Materials and methods

### 2.1 Patient enrolment and study design

We retrospectively enrolled 80 patients with hematological disorders with suspected or confirmed infection who underwent blood mNGS testing at Anhui Children’s Hospital in China from September 2023 to February 2024. According to our inclusion/exclusion criteria, data from a total of 73 patients were included in the current study for analysis. The inclusion criteria are as follows (1): hematologic diseases (2); High suspicion of infection, such as: fever (>38 °C), symptoms of respiratory/gastrointestinal/urinary tract infection, etc.; (3) Other relevant conventional microbial tests, such as culture, G/GM testing, and polymerase chain reaction (PCR) were completed simultaneously within 7 days of blood mNGS testing. Exclusion criteria are as follows: (1) non-hematologic disease; (2) incomplete case information; and (3) lack of paired CMT. Hematologic diseases mainly include leukemia, lymphoma, multiple myeloma, aplastic anemia, etc (Wang et al., 2024). Non-hematologic diseases mainly include diseases other than hematologic ones. In addition, we collected the demographic characteristics of the patients, clinical history, laboratory tests, treatment procedures. Two experienced clinicians independently assessed the patient’s clinical symptoms, laboratory tests, CMT, and response to treatment for the final diagnosis of infection.

This study was approved by the Ethics Committee (EYLL-2023-016) of Anhui Children’s Hospital, China. The study is considered exempt from informed consent as it is a retrospective observational cohort study.

### 2.2 Conventional microbiological tests

The patient’s peripheral blood was collected for both blood mNGS testing and routine blood cultures. Clinicians selected patients for CMT based on their clinical symptoms, signs, and imaging findings. Based on the suspected site of infection, samples of alveolar lavage fluid, sputum, cerebrospinal fluid, bone marrow, urine, as well as from PICC and ROPT, were collected for culture, smear, 1,3-β-d-glucan (G test), galactomannan (GM test), enzyme-linked immunospot (T-SPOT), and PCR detection (for *cytomegalovirus*, *Epstein-Barr virus*, *Mycoplasma pneumoniae*, *influenza A virus*, and *influenza B virus*).

### 2.3 mNGS sequencing and analysis

For nucleic acid extraction, the blood samples were centrifuged at 1900 ×g and 4°C for 10 minutes to obtain plasma for subsequent processing. Plasma cell-free DNA (cfDNA) was extracted using the PathoXtract^®^ Cell-Free Nucleic Acid Kit (WYXM03010S, WillingMed Corp, Beijing, China). RNA extraction was performed using the PathoXtract^®^ Virus Viral DNA/RNA Co-Extraction Kit (WYXM03009S, Microrock Medicine), both of which were performed according to the protocol. DNA and RNA from the same sample were pooled and reverse transcribed using the SuperScript^®^ Double-Stranded cDNA Synthesis Kit (11917020, Invitrogen) to reverse transcribe RNA into cDNA (Chen et al., 2024a; Xu et al., 2024).

The cfDNA samples were then processed for library construction using KAPA Hyper Prep Kits (KK8504, KAPA, Kapa Biosystems, Wilmington, MA, United States) following the manufacturer’s instructions. Sequencing was performed using a 50 bp single-ended method on the MGISEQ-200 platform (MGI Technology) (Kang et al., 2024). No-template control (NTC) and was set for each sequencing run to control the effects of contaminating DNA.

Bioinformatics analysis was performed as previously described (Chen H. et al., 2024). Trimmomatic v0.40 was designed to eliminate low-quality readings, contaminated joints, duplicate readings, and readings shorter than 36 bp (Bolger et al., 2014). The readouts were compared to the human reference genome GRCh38 (hg38) by using Bowtie2 software to remove the human sequence (Langmead and Salzberg, 2012). For taxonomic classification and identification of microbial reads, we utilized Kraken2 with non-redundant nucleotide sequences database of National Center for Biotechnology Information (NCBI) (Wood et al., 2019). Pathogen positivity is determined using a reading per ten million (RPTM) value. The viruses needed to reach an RPTM threshold of ≥3 to be considered positive; For bacteria and fungi, this threshold was ≥8. When the RPTM value was ≥1, specific pathogens, including *cryptococcus* and *mycobacteria*, were identified as positive (Wang et al., 2024).

### 2.4 Clinical impact of mNGS on treatment management

Based on the mNGS results, the clinical impact of mNGS on the treatment was assessed as positive, negative, and no effect (Xu et al., 2023; Zhu et al., 2023). Positive effects suggest that mNGS can be useful in antibiotic adjustment or confirm empiric regimens. Negative effects mean unnecessary treatment. No effect indicates that treatment has not been adjusted despite a negative or positive mNGS result, or that the patient has been discharged or died by the time mNGS reports are available.

### 2.5 Statistics analysis

Independent variables were expressed as counts and percentages. Continuous variables were presented as medians with interquartile ranges (IQR). The chi-square test was used for the analysis of categorical variables. Data analysis was performed using SPSS 26.0 software (IBM, United States). A p-value of less than 0.05 were considered statistically significant.

## 3 Results

### 3.1 Patient characteristics

In this study, a total of 73 children with hematologic disorders were included, including 42 males (57.53%) and 31 females (42.47%), with a median age of 6 years. They were classified into infected (n=64) and non-infected groups (n=9) based on their final clinical diagnosis, which was mainly determined by clinical symptoms, microbial test results, laboratory indexes, imaging features, and clinical treatment. Hematologic disorders included 13 cases (17.81%) of acute myeloid leukemia (AML), 37 cases (50.68%) of acute lymphoblastic leukemia (ALL), 7 cases (9.59%) of neuroblastoma (NBL), 3 cases (4.11%) of myelodysplastic syndromes (MDS), 2 cases (2.74%) of hemophagocytic Lymphohistiocytosis (HLH), 2 cases (2.74%) of Non-Hodgkin Lymphoma (NHL), 5 cases (6.85%) of aplastic anemia (AA) or other types of anemia, and 4 cases (6.25%) of other hematologic diseases. Of these patients, 66 (90.41%) were immunosuppressed, mainly due to chemotherapy for malignancy. Based on the final clinical diagnosis, the most common site of infection in infected patients was co-infection of the blood and lungs (43.75%), followed by blood-only infections (29.69%) and lung-only infections (15.63%). Overall, blood was the most common infection site in the patients in this study. In infected patients, the CRP index was significantly higher than that in non-infected patients (52.68 vs 3.44 mg/L, *P*=0.020) (**Table 1**). Among the non-infected patients, 77.78% were immunosuppressed, which was lower than in infected patients, but there was no statistically significant difference. There were no statistically significant differences in the other baseline data.

**Table 1:** Demographic and clinical characteristics in this study.

Demographics	Case (n=73)	Infected Group (n = 64)	Non-infected Group (n =9)	P value
**Age, years (M, IQR)**	6 (3-10)	7 (3-11)	6 (5-6)	0.657
**Male (n, %)**	42 (57.53%)	35 (54.69%)	7 (77.78%)	0.286
Site of infection				
Blood	19 (26.00%)	19 (29.69%)	—	
Lung	10 (13.70%)	10 (15.63%)	—	
Upper respiratory tract	2 (2.74%)	2 (3.13%)	—	
Blood+Lung	28 (38.36%)	28 (43.75%)	—	
Blood+Upper respiratory tract	2 (2.74%)	2 (3.13%)	—	
Blood+Abdomen	1 (1.37%)	1 (1.56%)	—	
Blood+Urinary tract	1 (1.37%)	1 (1.56%)	—	
Blood+Lung+Skin and soft tissues	1 (1.37%)	1 (1.56%)	—	
Hematologic diseases				
AML	13 (17.81%)	10 (15.63%)	3 (33.33%)	0.195
ALL	37 (50.68%)	35 (54.69%)	2 (22.22%)	0.085
NBL	7 (9.59%)	6 (9.38%)	1 (11.11%)	>0.999
MDS	3 (4.11%)	3 (4.69%)	0 (0.00%)	>0.999
HLH	2 (2.74%)	1 (1.56%)	1 (11.11%)	0.234
NHL	2 (2.74%)	2 (3.13%)	0 (0.00%)	>0.999
AA/Other anemias	5 (6.85%)	3 (4.69%)	2 (22.22%)	0.112
Other	4 (6.25%)	4 (6.25%)	0 (0.00%)	>0.999
**Immunosuppressive state**	66 (90.41%)	59 (92.19%)	7 (77.78%)	0.205
Laboratory examination				
WBC, 10^9/L	0.92 (0.20-2.69)	0.78 (0.18-2.19)	0.78 (0.19-2.18)	0.579
NE, 10^9/L	0.32 (0.01-1.73)	0.40 (0.01-1.67)	0.11 (0.07-0.58)	0.19
LY, 10^9/L	0.47 (0.12-1.08)	0.41 (0.10-0.84)	0.58 (0.09-1.19)	0.904
PLT, 10^9/L	67.50 (36.50-131.00)	62.50 (34.50-131.00)	83.00 (47.00-128.00)	0.709
CRP, mg/L	31.32 (5.56-99.38)	52.68 (14.01-114.41)	3.44 (0.70-27.76)	0.020*
PCT, ng/L	0.2 (0.10-0.46)	0.20 (0.10-0.46)	0.15 (0.12-0.33)	0.517

AML, acute myeloid leukemia; ALL, acute lymphoblastic leukemia; NBL, Neuroblastoma; MDS, myelodysplastic syndromes; HLH, Hemophagocytic Lymphohistiocytosis; NHL, Non-Hodgkin Lymphoma; AA, aplastic anemia; WBC, white blood cell count; NE, neutrophil count; LY, lymphocyte count; PLT, platelet; CRP, C-reactive protein; PCT, Procalcitoni; —, a site of infection not present in a patient who is eventually diagnosed as non-infectious. * *P*<0.05.

### 3.2 Comparison of the diagnostic performance of mNGS and CMT

**Figure 1:**
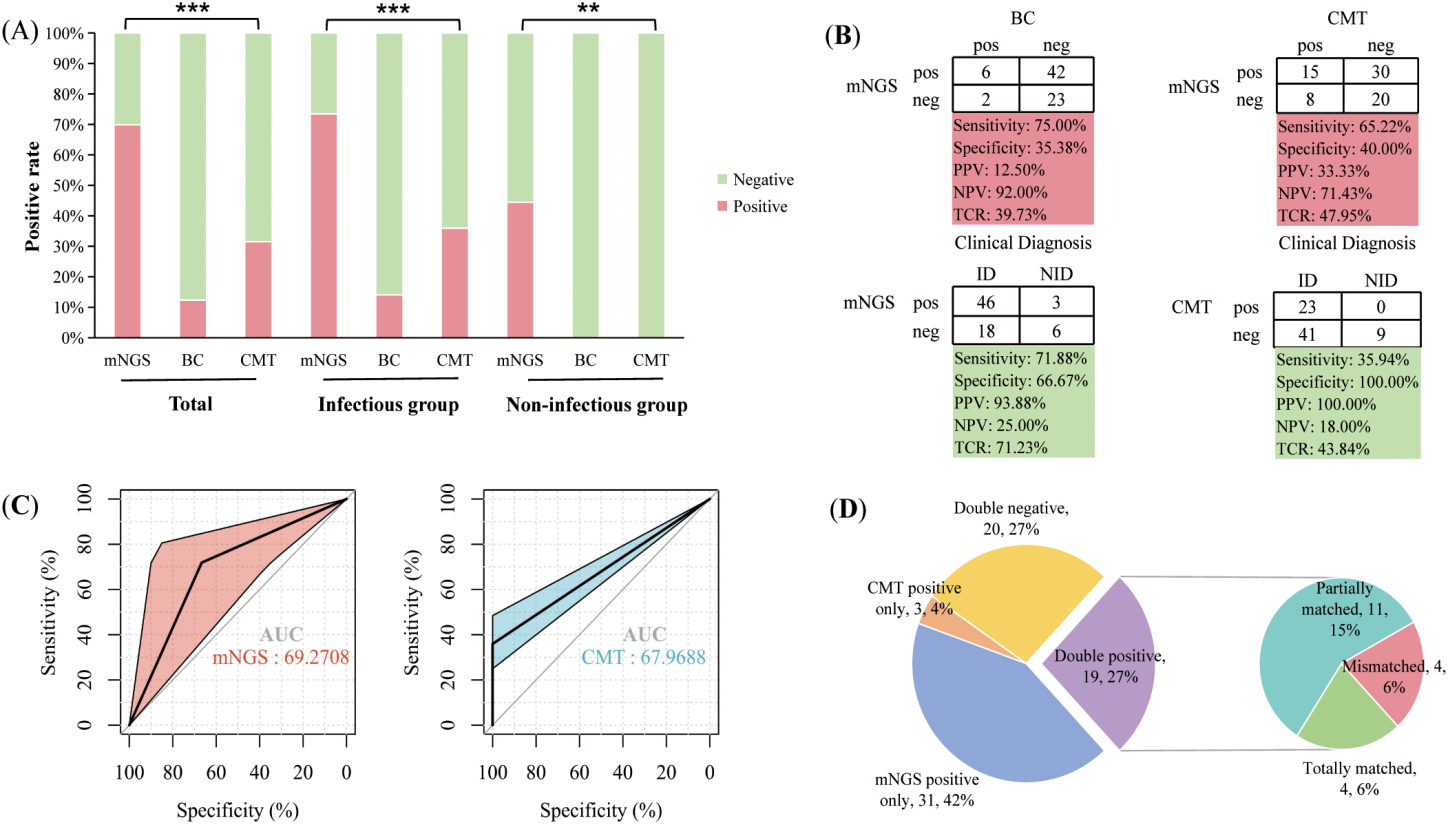
Comparison of the performance of plasma mNGS with blood culture (BC) and conventional microbial tests (CMT). **(A)** Positivity rates of different tests in total patients, as well as in infected and non-infected groups, where red indicates a positive proportion and green indicates a negative proportion; **(B)** Comparison of assay performance, including sensitivity, specificity, positive predictive value (PPV), negative predictive value (NPV) and total coincidence rate (TCR); **(C)** ROC curves of mNGS and CMT assays in detecting clinical infections; **(D)** Concordance analysis of mNGS and CMT results. **P<0.01, ***P<0.001.

In patients with hematologic disease, the positive rate of plasma mNGS was significantly higher than that of BC (69.86% vs 12.33%, *P* < 0.001) and CMT (69.86% vs 31.51%, *P* < 0.001). According to the final clinical adjudgment, the positive rate of plasma mNGS in the infected group was also significantly higher than that of BC (73.44% vs 14.06%, *P* < 0.001) and CMT (73.44% vs 35.94%, *P* < 0.001). In the non-infected group, plasma mNGS detected 44.44% of false-positive results (**Figure 1A**). A positive BC is often considered the “gold standard” for testing blood infections. When BC results were used as a reference, the sensitivity of plasma mNGS detection was 75%, with a specificity of 35.38%. When CMT results were used as the reference, the sensitivity and specificity of plasma mNGS were 65.22% and 40%, respectively. Using the final clinical diagnosis of infection as the gold standard, the sensitivity and specificity of plasma mNGS were 71.88% and 66.67%, respectively, with an overall accuracy of 71.23%. The sensitivity of CMT was 35.94%, while its specificity was 100%, resulting in an overall accuracy of 43.84%. The detection sensitivity (true positive rate) and overall accuracy of plasma mNGS were significantly higher than those of CMT (*P* < 0.001) (**Figure 1B**). However, despite the high sensitivity of plasma mNGS in detecting early infections, its false-positive rate was higher than that of CMT (33.33% vs. 0.00%, *P* = 0.205). For the final clinical diagnosis of infection, the AUC of the mNGS assay was 69.27, indicating high sensitivity and moderate specificity (**Figure 1C**). In contrast, the AUC of the CMT assay was 67.07, reflecting higher specificity. Consequently, mNGS has a slight advantage in sensitivity, while CMT performs better in specificity.

To compare the concordance between plasma mNGS and CMT. 26.03% (19/73) of patients were positive for both mNGS and CMT. 27.40% (20/73) of patients were negative. Positive pathogens were acquired by CMT alone in 4.11% (3/73) of patients and positive pathogens by plasma mNGS only in 42% of patients. Among the 19 double-positive patients, 21.05% (4/19) of patients had exact mNGS and CMT results, 57.89% (11/19) of patients were partially matched, but 21.05% (4/19) of patients were completely unmatched (**Figure 1D**). The main reason for the complete mismatch between the two methods is that plasma mNGS detects more bacteria, while CMT detects more viruses or mycoplasma by nucleic acid PCR.

### 3.3 Pathogen spectrum in patients with infectious diseases

**Figure 2:**
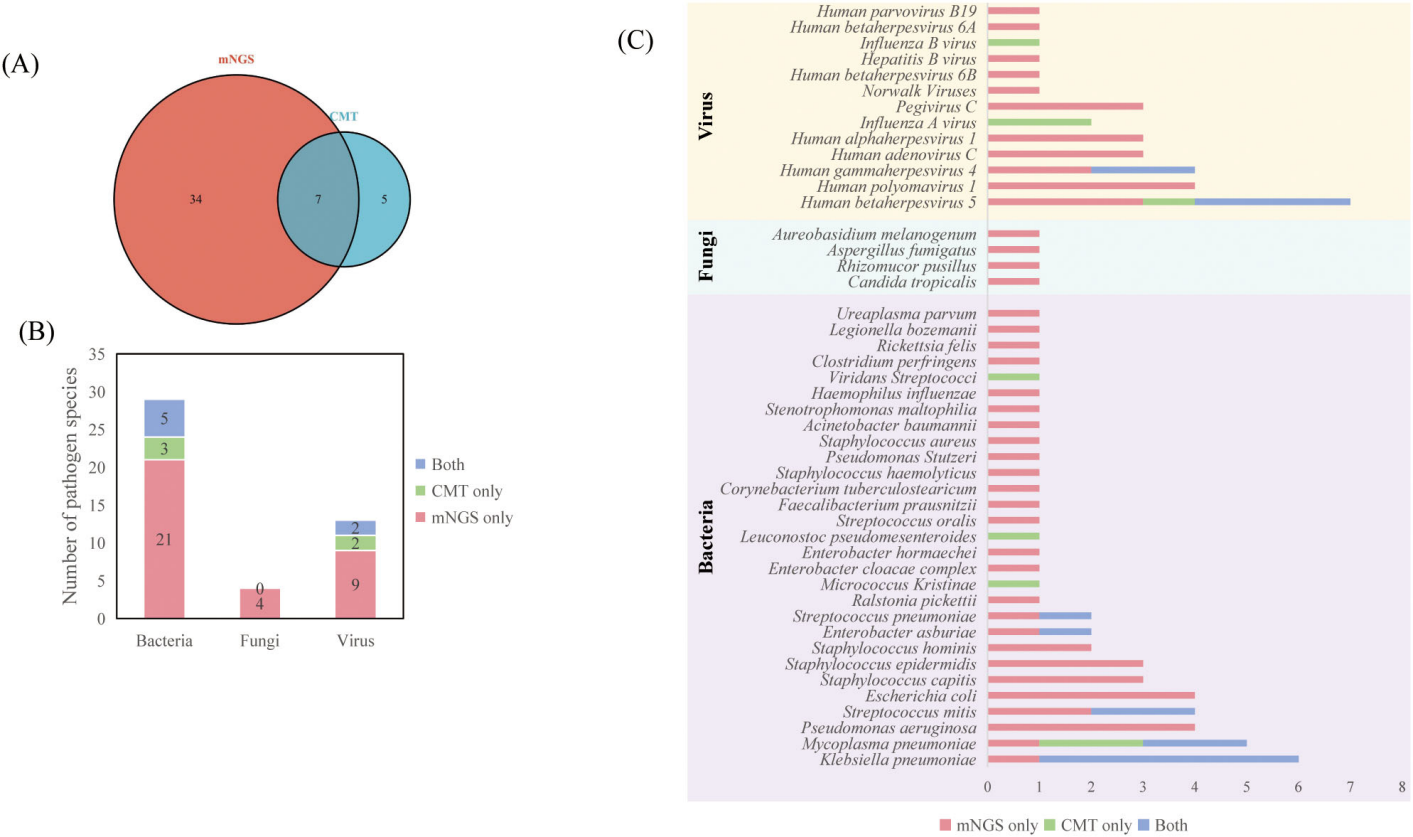
Distribution of pathogens detected by mNGS and CMT in infected patients. **(A)** Venn diagram showing the number of pathogens detected by mNGS and CMT, where red represents the pathogens detected by mNGS and blue represents those detected by CMT; **(B)** Types of pathogens detected by mNGS and CMT; **(C)** Pathogen spectrum for mNGS and CMT detection.

Plasma mNGS generates additional pathogen detection. In patients finally identified with infectious diseases, a total of 14,867,474 (11,757,006–19,357,164) sequences were obtained from plasma mNGS, including 14,500,279 (11,462,950–18,642,222) sequences aligned to the human genome and 1,981 (594–5,554) sequences aligned to the pathogenic microbial database. Among the sequences aligned to pathogenic microorganisms, 82.59% (48.40%–91.49%) were bacterial, 0.52% (0.16%–1.57%) were fungi, and 0.34% (0.00%–3.73%) were viruses. A total of 46 pathogens were detected in all infected patients, of which 34 pathogens were detected only in plasma mNGS. 5 pathogens were detected only by CMT (**Figure 2A**). 7 pathogens were detected by mNGS and CMT, including five bacteria and two viruses. mNGS detected most bacteria (26/29) and viruses (11/13), while fungi were detected only by mNGS (**Figure 2B**).

The most common bacteria detected in infected patients were *Klebsiella pneumoniae* and *Mycoplasma pneumoniae*. The viruses were *human betaherpesvirus* 5 (CMV), *human gammaherpesvirus* 4 (EBV), and *human polyomavirus 1* (BKV). The most common bacteria detected in CMT were *Klebsiella pneumoniae* and *Mycoplasma pneumoniae*, and the viruses were CMV, EBV and *Pegivirus* C. The most common bacteria detected by plasma mNGS were *Klebsiella pneumoniae*, *Pseudomonas aeruginosa*, *Streptococcus mitis* and *Escherichia coli*, and the most common viruses were CMV, EBV, and BKV. In addition, *Candida tropicalis*, *Rhizomucor pusillus*, *Aspergillus fumigatus*, and *Aureobasidium melanogenum* were only detected by mNGS (**Figure 2C**).

### 3.4 Impacts of plasma mNGS on clinical treatment

Among the 73 patients with suspected infection, plasma mNGS had a positive effect on the treatment of 65.75% (48/73) of patients (**Table 2**). 37 of these patients had their antibiotics adjusted based on mNGS results, and the remaining 11 patients, although not adjusted, had positive mNGS results that helped determine that the pathogen had been covered by empiric antimicrobial therapy. Plasma mNGS had no effect on 34.25% of patients, mainly focusing on negative plasma mNGS results. Plasma mNGS did not have a negative effect in this study. The positive effect of plasma mNGS in infected patients is higher than that in non-infected patients, and it is mainly used for the adjustment of antimicrobial regimens. In non-infected patients, the positive effect of mNGS is mainly manifested by the de-escalation or discontinuation of antibiotics, which helps to rule out infection.

**Table 2:** Clinical impact of plasma cfDNA mNGS on the treatment of infected patients.

Clinical impact	Grade	Description	Case number, n (%)	Infectious disease, n (%)	Non-infectious disease, n (%)	P value
Positive	M1	Initialization of the appropriate antibiotics treatment	8 (10.96%)	8 (12.50%)	0 (0.00%)	0.055
	M2	Antibiotic escalation	11 (15.07%)	11 (17.19%)	0 (0.00%)	
	M3	Antibiotic de-escalation	18 (24.66%)	15 (23.44%)	3 (33.33%)	
	M4	Confirmed empirical treatment	11 (15.07%)	11 (17.19%)	0 (0.00%)	
No effect	M5	No adjustment in treatment while the result was positive	9 (12.33%)	6 (9.38%)	3 (33.33%)	0.055
	M6	The patient was discharged or dead	0 (0.00%)	0 (0.00%)	0 (0.00%)	
	M7	No adjustment in treatment while the result was negative	16 (21.92%)	13 (20.31%)	3 (33.33%)	
Negative	M8	NGS led to unnecessary treatment	0 (0.00%)	0 (0.00%)	0 (0.00%)	ns

### 3.5 Variations in laboratory indices across different mNGS results

**Figure 3:**
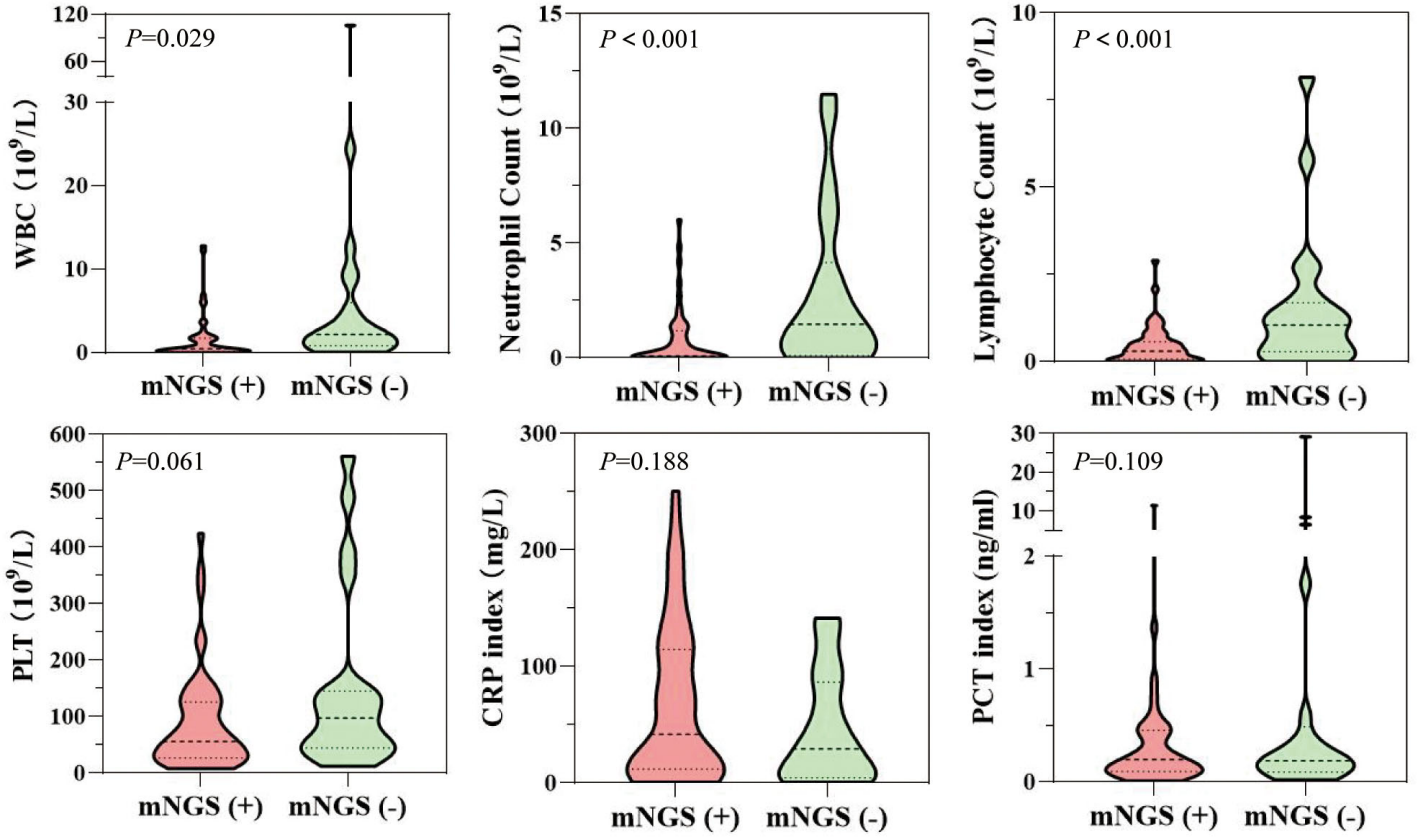
Differences in laboratory indices (routine blood indices synchronized with plasma mNGS or 48-hour detection) between patients in the mNGS-positive and mNGS-negative groups.

A positive result of plasma mNGS contributes to clinical adjudication in patients with suspected infection. Patients in the mNGS-positive group had significantly lower white blood cell count (WBC) (P=0.029), neutrophil count (NE) (P<0.001), and lymphocyte count (LY) (P<0.001) compared to those in the mNGS-negative group. No significant differences were observed between the two groups in terms of C-reactive protein (CRP) and procalcitoni (PCT) indexes (**Figure 3**).

## 4 Discussion

Children with hematologic diseases tend to be at increased risk of infection due to weakened immune systems. In particular, patients with hematologic malignancies are more likely to cause complex and severe infections during treatment (Fu et al., 2022). In this study, leukemia was the most common type of disease in pediatric patients, mainly acute lymphoblastic leukemia and acute myeloid leukemia. Previous studies have shown that infection-related mortality is a major factor influencing the outcome of pediatric acute leukemia treatment (Villeneuve and Aftandilian, 2022). 90.41% of patients were immunosuppressed, and immunosuppressed patients were more likely to be infected by unusual pathogens and more likely to develop severe co-infections (Casto et al., 2022). In patients who are eventually clinically diagnosed as infectious, the most common site of infection was blood, followed by the lungs. Bloodstream infections and lung infections are also the most common sites of infection in patients with hematologic diseases, with a mortality rate of up to 37.1% associated with bloodstream infections (Yin et al., 2021). Therefore, it is important to identify pathogens quickly, accurately, and effectively and to initiate targeted antibiotic therapy.

Pathogen detection using non-invasive samples of plasma cfDNA reduces invasive trauma in children. In this study, the positive rate of plasma cfDNA mNGS was significantly higher than that of the gold standard “BC” and CMT by about 38%-57%, especially in infectious patients. A number of hematological studies have reported that the positive rate of BC is low, only 10%-13%, and in this study, the positive rate of BC is only 12.33%, which is also similar to previous studies, although BC is an important reference for clinical diagnosis, it is difficult to detect pathogens quickly and broadly by blood culture alone (Nannan Panday et al., 2019; Zhang et al., 2023). The significant advantages of mNGS in detection have also led to its recommendation as an additional detection method for first-line detection tools (Li et al., 2021). Compared to BC, mNGS achieved a sensitivity of 75%. The sensitivity of mNGS was 71.88% as a reference to the final clinical diagnosis. This is similar to a previous systematic review of infection in patients with hematologic disorders, and plasma mNGS has significant advantages over CMT in detecting microbial infections (Chen et al., 2024c). Previous studies have shown that in adult patients with hematologic disorders, the overall coincidence rate of blood mNGS was 51.02% when CMT was used as a reference (Hao et al., 2022). Although the patient population in our study was children, the overall coincidence rate of mNGS compared to CMT was similar to that of adults (47.95%). However, when the final clinical diagnosis is used as the criterion, mNGS shows lower specificity, although it has higher sensitivity than CMT. This differs from previous studies, which may be due to the fact that in our study, CMT included culture, smear, G/GM testing, and targeted PCR testing for pathogens. In contrast, the broad spectrum of mNGS leads to false-positive results, decreasing its specificity. In particular, most of the false-positive results detected in this study were herpes viruses identified by mNGS. Low abundance of DNA viruses is also a major cause of false-positive results in patients with hematologic disorders (Wu et al., 2024). It is important to note that the final results of mNGS still need to be adjudicated by professional clinicians to avoid interference from non-pathogenic microorganisms.

mNGS technology has identified a wider range of infectious pathogens in patients with hematological disorders. Forty-one pathogens were detected by mNGS, compared to only 12 by CMT. Patients with blood malignancies have an increased risk of bacterial infections after chemotherapy (Thacker et al., 2014; Wu et al., 2024). Similar to previous reports, a large number of bacteria were detected in our study (Chen et al., 2024b). *Klebsiella pneumoniae* and *Mycoplasma pneumoniae* were the most common. They are also the most common pathogens detected by CMT. *Klebsiella pneumoniae* is a common microorganism for infection in people with hematological diseases. But it’s worth noting that more *Mycoplasma pneumoniae* was detected in our study, which differs from the epidemiology of patients with hematological diseases. We reviewed the clinical information of the patients and found that these children all had infections that occurred during the winter months, so the detection of *Mycoplasma pneumoniae* may be associated with seasonal infections in children. For the detection of viruses, CMV and EBV are the most common viruses detected, which is basically consistent with the pathogen spectrum in previous studies (Zhang et al., 2022; Chen et al., 2024b). In addition, mNGS was more sensitive in the detection of fungi, and the fungi in all 4 patients were detected by mNGS, while CMT was negative. Timely identification of fungal infections is essential for patients with hematological diseases, which also reflects the advantages of mNGS in the detection of fungi (Guo et al., 2022). Despite the broad spectrum and high sensitivity of mNGS in detecting pathogens, some pathogens are still detected only by CMT. We found that the main pathogens missed by mNGS were Mycoplasma pneumoniae and influenza viruses (A and B), which may be due to the fact that PCR targets Mycoplasma pneumoniae and influenza viruses (A and B) in CMT assays. Several previous studies have also shown that targeted PCR has higher detection sensitivity for specific pathogens compared to mNGS (Hu et al., 2021). Therefore, supplementing with plasma mNGS can provide valuable insights when infection is suspected in patients with hematologic disorders at an early stage.

Plasma mNGS had a positive effect on the treatment of 65.75% of patients. This is mainly focused on reducing unnecessary drugs based on the results of mNGS. Unnecessary antibiotic treatment will lead to the development of drug resistance and increase the medical burden of patients (Wang et al., 2024). 26.03% of patients adjusted or increased the use of targeted antimicrobial drugs according to mNGS results. Although clinicians always empirically use broad-spectrum antibiotics when infection is suspected, mNGS can help target antibiotics and improve clinical symptoms in patients (Chen et al., 2024c). However, in our study, there were still 34.25% of patients who were not affected by mNGS, and they mainly focused on the negative results of mNGS and did not provide a reference for the adjustment of antibiotics. In addition to this, mNGS also had a positive effect on 33.33% of non-infected patients, which led to the discontinuation of antibiotics due to the exclusion of infection. mNGS had a significant positive effect in patients with suspected infection in the early stage, benefiting most patients.

Patients with blood disorders are more likely to develop neutropenia after clinical treatment (chemotherapy, transplantation, etc.), and patients are more likely to develop infections. In our study, patients with neutropenia were more likely to have positive results for mNGS testing, which is consistent with previous reports. Previous reports have demonstrated that pediatric patients with hematologic malignancies and neutropenia are more susceptible to bacterial infections, which was also confirmed in our study, where more bacterial species were detected by blood mNGS (Wu et al., 2024). In addition to this, we also found that lower WBC and lymphocyte counts made it easier to obtain positive results for mNGS. This may be due to the fact that lower white blood cell and lymphocyte counts increase the risk of severe infection, meaning that patients are more critically ill, and mNGS tests are more sensitive and more likely to obtain positive results in patients with severe disease (Chang et al., 2023). Additionally, pediatric patients with low lymphocyte counts are more susceptible to CMV infection (Zhan et al., 2022). In our study, CMV was also the most commonly detected virus. These results provide valuable reference for using mNGS detection in patients with suspected infection at an early stage.

However, our research also has some limitations. First, because this is a retrospective study, we did not collect samples for mNGS on the same date, resulting in the majority of patients receiving empiric antimicrobial therapy prior to mNGS testing. This may have an impact on the results of mNGS and CMT. Second, although we have a verdict on clinical infection, there is still a lack of criteria for each pathogen to determine whether they are clinically relevant or not, so the final verdict is still determined by the subjective experience of the clinician. Finally, this is a small retrospective study, the results may be biased and a prospective study of large-scale, multicenter subjects is still needed to further validate the relevant findings.

## 5 Conclusion

This study demonstrated the value of plasma mNGS in the diagnosis of suspected infection in children with blood disorders in the early stage. mNGS is far superior to BC and CMT in detecting infections and helps rule out infections. mNGS detection at an early stage of infection helps in the identification of pathogens and the precise treatment of antimicrobial drugs, thereby improving patients’ symptoms and reducing mortality.

## Data Availability

The data presented in the study are deposited in the SRA (https://www.ncbi.nlm.nih.gov/bioproject/PRJNA1227282/) repository, accession number PRJNA1227282.
